# Carotid cavernous fistula masquerading as delayed suprachoroidal hemorrhage after trabeculectomy

**DOI:** 10.3205/oc000071

**Published:** 2017-08-25

**Authors:** Mukesh Jain, Md. Shahid Alam, Bipasha Mukherjee, Rajiv Raman

**Affiliations:** 1 Shri Bhagwan Mahavir Vitreoretinal Services, Sankara Nethralaya, Chennai, India; 2 Department of Orbit Oculoplasty Reconstructive and aesthetic services, Sankara Nethralaya, Chennai, India

**Keywords:** carotid cavernous fistula, delayed suprachoroidal hemorrhage, trabeculectomy

## Abstract

**Objective:** Carotid cavernous fistulae (CCFs) are abnormal communications between the cavernous sinus and the carotid arterial system. Based on the etiology, CCFs can be traumatic, spontaneous and rarely iatrogenic. We report an interesting case of new onset CCF associated with shallow choroidal detachment after trabeculectomy surgery.

**Method:** Observational case report

**Result:** A 69-year-old male patient presented with complain of proptosis, congestion, and gross diminution of vision in the left eye following trabeculectomy elsewhere. Delayed suprachoroidal hemorrhage was diagnosed by the primary physician and the patient was on oral steroids. On USG-B scan, choroidal detachment and a dilated superior ophthalmic vein were seen. A digital subtraction angiogram showed type D CCF. In view of nil visual prognosis, he was treated conservatively and was referred to a neuroradiologist for further management.

**Conclusion:** CCFs have been rarely reported after intraocular surgeries. Till date, there is one case report of CCF complicating cataract surgery. Interestingly, CCFs has not been reported complicating trabeculectomy surgery. Dural CCFs although uncommon should be considered a close differential of hemorrhagic choroidal detachment, a relatively common complication following intraocular surgery.

## Introduction

A carotid cavernous fistula (CCF) is an abnormal connection between the carotid arterial system and the cavernous sinus. CCFs can be classified based on anatomy (direct or dural), etiology (traumatic or spontaneous) or blood flow velocity (high or low). Although relatively uncommon, CCFs have been reported following various diagnostic and therapeutic procedures of head and neck. However, CCFs have been rarely reported after intraocular surgeries. Till date, there is one case report by Nagaki et al. of CCF complicating cataract surgery [1]. We report an interesting case of new onset CCF associated with shallow choroidal detachment after trabeculectomy surgery.

## Case description

A 69-year-old male patient presented with the chief complain of prominence of the left eye associated with periocular swelling, redness, and gross diminution of vision of 1 month duration.

He was a known case of primary open angle glaucoma on anti-glaucoma medication. Trabeculectomy in the left eye was performed, after which his symptoms developed on post-operative day 3. He was diagnosed to have hemorrhagic choroidal detachment and was treated with steroids with little benefit. He was a known case of diabetics and hypertension on regular medical therapy.

On examination, his best corrected visual acuity was 6/9 and N6 in the right eye and no light perception (PL negative) in the left eye. Extraocular movements were full and free in the right eye but were limited in all directions of gazes in the left eye. There was periorbital edema and near total ptosis in the left eye. Hertels exophthalmometry revealed reading of 21 mm in the right and 30 mm in the left eye, with a base reading of 110. 

**Figure 1:**
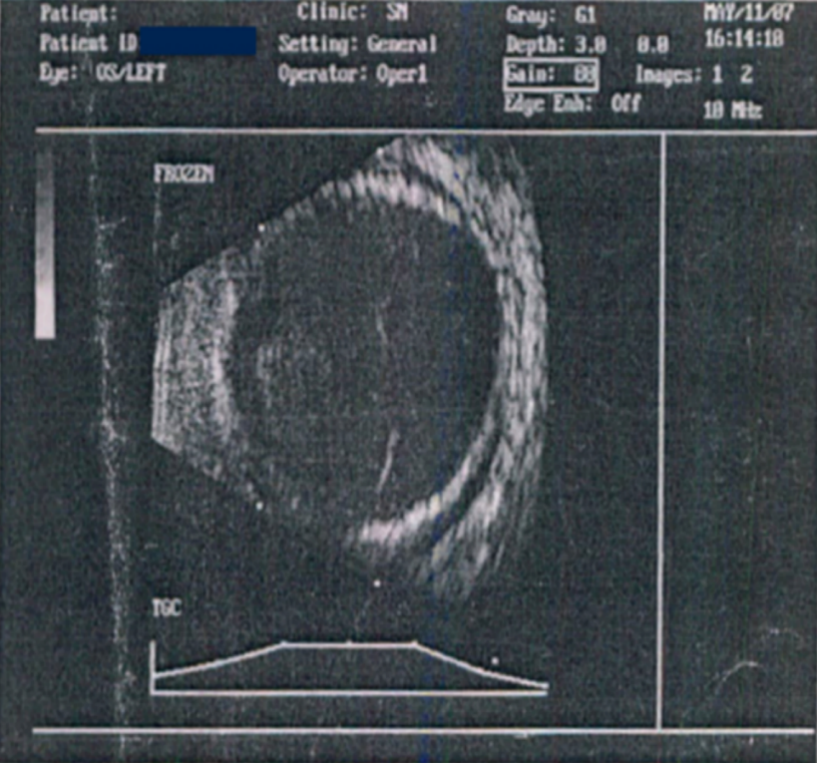
USG B-scan of the left eye showing shallow 360 degree of serous choroidal detachment.

Slit lamp examination of the right eye was normal except for the early cataractous changes in the lens. In the left eye, there was conjunctival congestion, chemosis associated with dilated episcleral vessels. Anterior chamber was flat with total cataract. Intraocular pressure in OD was 24 mm of Hg and 30 mm of Hg in the left eye by Goldman Applanation Tonometry. Fundus examination revealed a vertical cup: disc ratio (C:D ratio) to be 0.7 with neuroretinal rim thinning in the right eye with the rest of the fundus being normal. No view of the fundus could be obtained in the left eye. USG-B scan was done which revealed thickened extraocular muscles with optic nerve head cupping, shallow 360 degree choroidal detachment with no evidence of mass lesion, and a dilated superior ophthalmic vein on the left side (Figure 1 (Fig. 1)).

**Figure 2:**
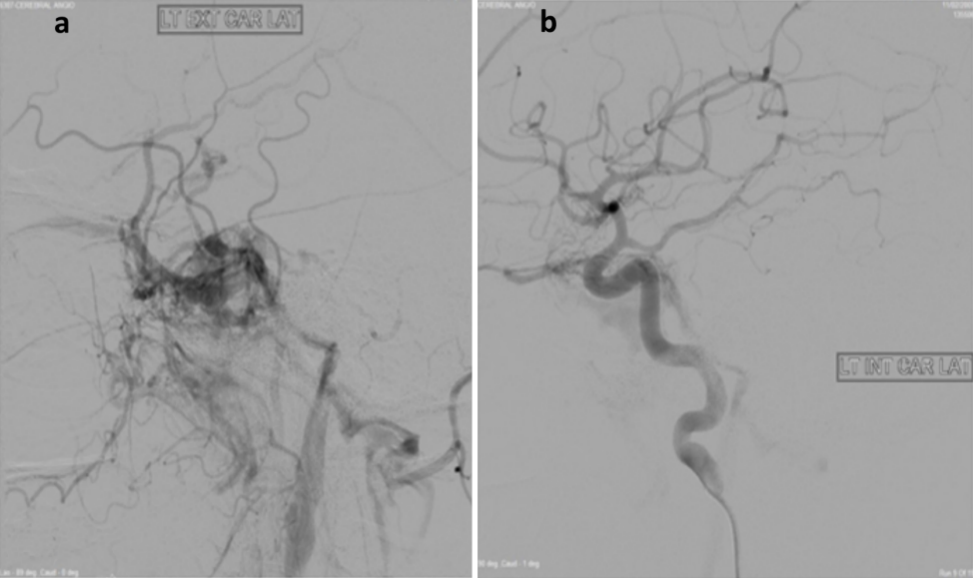
Digital subtraction angiogram confirming an indirect type D carotid cavernous fistula with communication between cavernous sinus and the dural branches of left (a) external carotid artery (b) and internal carotid artery.

Based on the clinical signs, symptoms, and initial investigations, carotid cavernous fistula was suspected and further investigated. A MRI was performed which showed prominent left cavernous sinus with abnormal flow with early opacification of the left superior ophthalmic vein on dynamic contrast and enhanced MR angiogram. A four vessel angiogram revealed type D CCF (Figure 2 (Fig. 2)).

**Figure 3:**
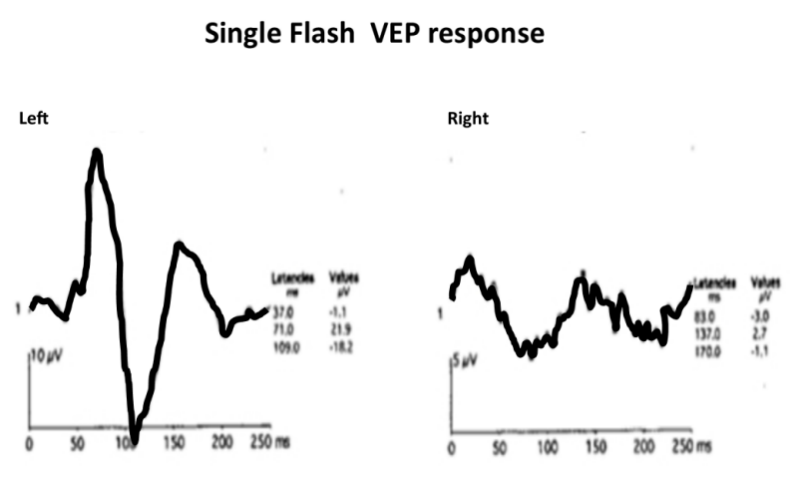
Visual evoked potential with normal P100 amplitude and latency in the right eye and grossly diminished P100 amplitude and latency in the left eye.

Visual evoked potential was done which revealed normal P100 amplitude and latency in OD and grossly diminished P100 amplitude and latency in OS (Figure 3 (Fig. 3)). Perimetry was performed in OD to document the visual field loss.

In view of nil visual prognosis in OS, he was advised conservative management and for OD was prescribed anti-glaucoma medication and regular follow-up in the glaucoma clinic. He was referred to an interventional radiologist for opinion and further management as required. The patient subsequently was lost to follow-up.

## Discussion

We report an interesting case of a 69-year-old male patient who developed proptosis, congestion and gross diminution of vision with shallow choroidal detachment following trabeculectomy. Interestingly, he was misdiagnosed as hemorrhagic choroidal detachment and treated conservatively with steroids. We carefully re-evaluated him and he was found to have type D CCF. Dural CCF and non-appositional hemorrhagic choroidal detachment have quite similar clinical profiles. It can be quite challenging to differentiate the two in the postoperative period especially when the latter is a well known complication and the former has not been reported in the literature.

Delayed suprachoroidal hemorrhage after intraocular surgery is characterized by acute onset severe pain, diminution of vision, shallow anterior chamber, and raised intraocular pressure. The reported incidence of delayed suprachoroidal hemorrhage after glaucoma filtration surgery varies from 1.6% to 6.2%, depending on the type of surgery performed [2], [3], [4], [5], [6], [7], [8], [9]. Tuli et al. reported an incidence of 1.5% and 2.4% after trabeculectomy without antimetabolites and trabeculectomy with anti-metabolites, respectively [10]. Old age, myopia, aphakia, hypertension, high pre-operative intraocular pressure, vitrectomy, postoperative hypotony has been suggested as risk factors [2], [3], [4], [5], [6], [7], [8], [9], [10].

CCF can present with varied presentations: conjunctival chemosis, congestion, proptosis, ophthalmoplegia, double vision, orbital pain, bruit and diminution of vision. Because of the diverse clinical presentation with a long list of differentials, CCFs are misdiagnosed in many, leading to significant morbidity and mortality, including visual loss. Sutoh et al. described the significant remodeling of choroid in patients with CCFs [11]. Choroidal detachment and retinal detachment have been described in CCF patients in the literature [12], [13], [14]. Choroidal detachment in CCFs result from severe choroidal vascular congestion, stasis, and increased fluid transudation secondary to raised venous pressure.

History of hypertension, proptosis, dilated episcleral vessels, shallow serous choroidal detachment, dilated superior ophthalmic vein, and refractory to steroid therapy helped us to suspect CCF in this patient. Ordering appropriate investigations, MRI followed by angiography confirmed the diagnosis of type D in this patient.

It might be possible that the CCF was present before surgery and worsened following trabeculectomy. But this seems less likely in the absence of any history of proptosis, redness or diplopia prior to surgery. Even if so, it presented itself following glaucoma surgery and posed a diagnostic challenge. Wether it was a mere coincidence that CCF appeared following trabeculectomy or if trabeculectomy surgery contributed in any way to the occurrence, remains unanswered.

## Conclusions

In conclusion, it can be difficult to differentiate the two, delayed hemorrhagic choroidal detachment and new onset dural CCF, in postoperative period especially when the former is a well known complication. Nevertheless, a good history taking and meticulous clinical examination is necessary. Early diagnosis and appropriate specific treatment could have prevented the blindness in this case.

## Notes

### Competing interests

The authors declare that they have no competing interests.

### Acknowledgement

We would like to thank our colleagues in Shri Bhagwan Mahavir Vitreoretinal Services, Sankara Nethralaya, Chennai, for their support.
